# Efficacy of Solifenacin, Mirabegron, and Combination Therapy in Children With Daytime Urinary Incontinence (BeDry): Protocol for a Randomized Single-Blinded Controlled Trial

**DOI:** 10.2196/63588

**Published:** 2025-06-26

**Authors:** Ann-Kristine Mandøe Svendsen, Søren Hagstrøm, Kristina Thorsteinsson, Jason Van Batavia, Konstantinos Kamperis, Anne Estrup Olesen, Luise Borch

**Affiliations:** 1 Department of Pediatrics and Adolescent Medicine Gødstrup Hospital Herning Denmark; 2 NIDO Centre for Research and Education Gødstrup Hospital Herning Denmark; 3 Department of Clinical Medicine Aarhus University Aarhus Denmark; 4 Department of Pediatrics and Adolescent Medicine Aalborg University Hospital Aalborg Denmark; 5 Department of Clinical Medicine Aalborg University Aalborg Denmark; 6 Division of Urology Children's Hospital of Philadelphia Philadelphia, PA United States; 7 Department of Pediatrics and Adolescent Medicine Aarhus University Hospital Aarhus Denmark; 8 Aalborg University Hospital Department of Clinical Pharmacology Aalborg Denmark

**Keywords:** urinary incontinence, overactive bladder, solifenacin, mirabegron, children, teenager, pediatric, protocol, randomized controlled trial, pharmacotherapy, urotherapy, monotherapy, Denmark, outpatient clinics, multicenter

## Abstract

**Background:**

According to International Children’s Continence Society, the first-line treatment of children with daytime urinary incontinence is standard urotherapy, eventually followed by pharmacotherapy of anticholinergics. The effect of medical treatment is sparsely investigated and has been investigated primarily in nonrandomized trials.

**Objective:**

The primary objective of this trial is to evaluate if (1) combination therapy of solifenacin and mirabegron in low doses is superior to monotherapy of solifenacin in high dose and if (2) combination therapy of mirabegron and solifenacin in low doses is superior to monotherapy of mirabegron in high dose in the treatment of daytime urinary incontinence among children aged 5-14 years who are noncomplete responders to monotherapy of solifenacin in low dose or monotherapy of mirabegron in low dose. The secondary objective is to evaluate the treatment response of combination therapy of solifenacin and mirabegron in low doses, monotherapy in high doses, and monotherapy in low doses as supplementary comparisons. Additionally, the secondary objective is to evaluate the side effects, safety, and tolerability of the medical treatment as well as the effect of the treatment on the well-being and quality of life.

**Methods:**

Children aged 5-14 years diagnosed with daytime urinary incontinence refractory to standard urotherapy will be randomized 1:1:1:1 to 4 treatment groups. Initially, 2 groups will receive solifenacin 5 mg and 2 groups will receive mirabegron 25 mg. After 6 weeks, noncomplete responders will receive add-on treatments according to their primary randomization group. Group 1A will receive solifenacin 5 mg and add-on solifenacin 5 mg, group 1B will receive solifenacin 5 mg and add-on mirabegron 25 mg, group 2A will receive mirabegron 25 mg and add-on mirabegron 25 mg, and group 2B will receive mirabegron 25 mg and add-on solifenacin 5 mg. The total treatment period will be 18 weeks. The primary end point measure is treatment response assessed by change from visit 2 to the end of the study, according to the number of wet days per 7 days by DryPie.

**Results:**

The BeDry study was approved by Clinical Trials in the European Union on June 12, 2024. Recruitment began on June 27, 2024, and will continue until 236 patients are included, which is expected to occur by September 2026. As of April 2025, 75 participants are included.

**Conclusions:**

This trial has the potential to optimize the medical treatment of children with daytime urinary incontinence, shorten the treatment period, diminish the side effects, and minimize unnecessary medical expenses.

**Trial Registration:**

Clinical Trials in the European Union EUCT 2023-510187-13-00; https://tinyurl.com/2hva7ph8; ClinicalTrials.gov NCT06551246; https://clinicaltrials.gov/study/NCT06551246

**International Registered Report Identifier (IRRID):**

PRR1-10.2196/63588

## Introduction

### Background and Rationale

Daytime urinary incontinence (DUI) is affecting up to 1 out of 5 children aged 5-7 years [1-4] and 4.5% of the children and adolescents aged 11-16 years [3,4]. Urinary incontinence is usually a physically benign condition [2]. Nevertheless, it is associated with a considerable psychological burden, as it leads to poor self-esteem and quality of life among the affected children, leading to psychological failure to thrive [5,6].

The most common cause of DUI is an overactive bladder (OAB) [2,7]. The International Children’s Continence Society defines OAB as a condition with urinary urgency with or without DUI among children and adolescents without competitive pathology explaining the symptoms such as neurogenic detrusor overactivity or urinary tract infection [8].

Treatment often requires a multidisciplinary effort involving contributors in the health care system, home, and day care/school. The first-line treatment of urinary incontinence is urotherapy, aiming at improving the bladder reservoir function and the voluntary bladder control [2]. If urotherapy is insufficient for achieving continence, the second-line treatment in most clinical settings is addition of pharmacological treatment aiming at suppressing smooth bladder muscle contraction [2].

Solifenacin and mirabegron act on the bladder through different mechanisms. Solifenacin is an antimuscarinic agent that selectively inhibits M2/M3 muscarinic receptors in the bladder [2]. Mirabegron is a β3-adrenergic receptor agonist that stimulates β3 receptors in the detrusor muscle. While solifenacin decreases bladder contractions by inhibiting parasympathetic activity, mirabegron promotes bladder relaxation through sympathetic stimulation [9].

In Denmark, the current pharmacological therapy consists of an antimuscarinic, namely, solifenacin, or a β3-adrenoreceptor agonist, namely, mirabegron. If monotherapy of solifenacin or mirabegron is without sufficient effect, the child is treated with a combination of solifenacin and mirabegron. According to expert opinions, the current pharmacological treatment strategy in Denmark typically follows this sequence: initially, solifenacin is prescribed at 5 mg daily, with an increase to 10 mg depending on the therapeutic response. If monotherapy in high dose proves insufficient, add-on therapy with mirabegron at 25 mg daily is introduced and may be increased to 50 mg depending on the treatment effect. Occasionally, mirabegron monotherapy is used as an alternative second step before combination therapy. Solifenacin and mirabegron are widely used off-label in the pediatric population, and only a few studies have dealt with the efficacy and tolerability of these agents [10-14]. Only 1 study has evaluated combination therapy [14].

Since the pharmacological approach is widely used off-label in pediatric departments, an evidence-based treatment approach of pharmacological therapy is highly needed. This study aims to evaluate the efficacy, safety, and tolerability of solifenacin, mirabegron, and a combination of solifenacin and mirabegron in pediatric patients with DUI.

### Objectives

We hypothesize that combination therapy with solifenacin 5 mg and mirabegron 25 mg is effective in the reduction of DUI episodes over an 18-week treatment period compared to baseline and that combination therapy in low dose is superior to high dose of either solifenacin 10 mg or mirabegron 50 mg.

The primary objective is to evaluate the effect of high dose anticholinergic monotherapy (solifenacin 10 mg) compared to that of low dose combination therapy of anticholinergic and β3-adrenoreceptor agonist (solifenacin 5 mg and mirabegron 25 mg) and the effect of high dose β3-adrenoreceptor agonist monotherapy (mirabegron 50 mg) compared to that of low dose combination therapy of anticholinergic and β3-adrenoreceptor agonist (solifenacin 5 mg and mirabegron 25 mg) for the treatment of DUI in children aged 5-14 years who are noncomplete responders to standard urotherapy and to low dose anticholinergic or β3-adrenoreceptor agonist monotherapy.

The secondary objective is to compare the treatment response to combination therapy of solifenacin and mirabegron in low doses, monotherapy with solifenacin or mirabegron in high doses, and monotherapy with solifenacin or mirabegron in low doses. Additionally, the secondary objective is to evaluate the side effects, safety, and tolerability of the medical treatment as well as the effect of the treatment on the well-being and quality of life.

## Methods

### Study Design

**Figure 1:**
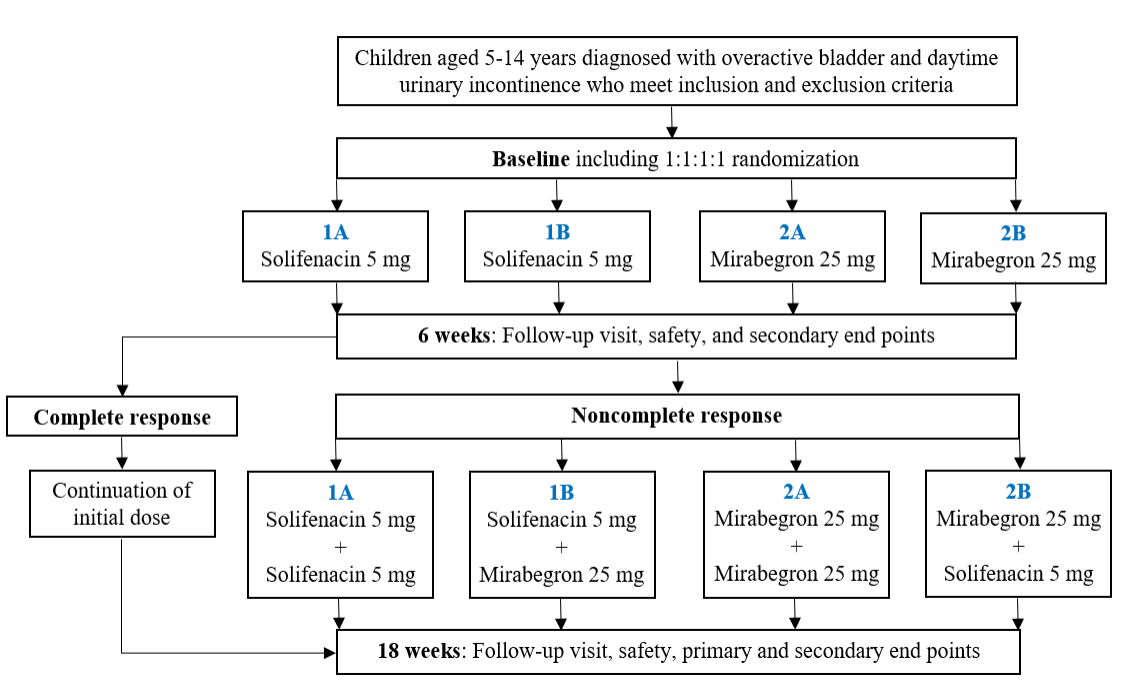
Study flowchart.

The BeDry study is designed as a multicenter, randomized, single-blind, controlled clinical trial. Included children will be randomized 1:1:1:1 to one of the 4 treatment groups. The study duration for each participant will be 18 weeks and encompasses 3 visits to the outpatient clinic. See Figure 1 for an overview of the study design. Children aged 5-14 years who are diagnosed with OAB and DUI will be included if they meet the criteria for inclusion. This trial is in accordance with the CONSORT (Consolidated Standards of Reporting Trials) checklist (Multimedia Appendix 1). This study is registered at the research inventory of the Regions of Denmark (1-16-02-210-24) and at Aarhus University (ARG-2024-731-23829). Recruitment will take place from pediatric departments at 5 outpatient clinics in Denmark: Aarhus University Hospital, Aalborg University Hospital, Regional Hospital Kolding, Regional Hospital Esbjerg, and Gødstrup Hospital.

### Eligibility Criteria

The inclusion and exclusion criteria for the participants in this study are provided in Textbox 1.

**Inclusion and exclusion criteria.**

**Inclusion criteria**

- The participants’ custody holders must voluntarily sign and date an informed consent prior to initiation of any study-specific procedures.
- Age 5-14 years (inclusive) at the time of inclusion.
- Overactive bladder as per International Children’s Continence Society criteria.
- At least 2 daytime urinary incontinence episodes per week.
- Inadequate effect of at least 4 weeks of urotherapy (nonpharmacological treatment).
- No previous treatment with solifenacin, mirabegron, or bladder/sphincter botulinum toxin injections.
- No current constipation as per Rome IV criteria or fecal incontinence (laxative treatment is accepted).
- Per the investigator’s judgment, the participant can swallow or can learn to swallow the study medication.

**Exclusion criteria**

- Inability of the patient or legal guardian to understand the Danish written and oral information.
- Known or suspected hypersensitivity to study medication.
- Any contraindication to the use of the study medication.
- Known urogenital anatomical abnormalities affecting lower urinary tract function.
- Known kidney or bladder stones.
- Known diabetes insipidus.
- Ongoing symptomatic urinary tract infection.
- Recurrent urinary tract infection or ongoing prophylactic antibiotic treatment.
- Known QTc prolongation, QTc >460 milliseconds, or risk of QTc prolongation (hypokalemia, exercise-induced syncope, or familial long QT syndrome).
- Other significant electrocardiogram abnormalities.
- Known hypertension.
- ≤3 daily voiding, evaluated by a 48-hour frequency-volume chart.
- Uroflowmetry is indicative of pathology other than overactive bladder (staccato-shaped, interrupted-shaped, or plateau-shaped curve).
- Postvoid residual urine >50 mL after double voiding.
- Dipstick hematuria (≥2+ erythrocytes) or macroscopic hematuria.
- Pregnancy or breastfeeding.
- Females of childbearing potential.
- Ongoing constipation according to Rome IV criteria, which is intractable to medication or fecal incontinence.
- Inability to swallow study medication.
- Use of any medication during the study period, except permitted medication.

### Intervention

Eligible children will be randomized 1:1:1:1 to one of the following 4 treatment groups.

1. Treatment group 1A: Solifenacin 5 mg (and if noncomplete response after 6 weeks, add-on solifenacin 5 mg).
2. Treatment group 1B: Solifenacin 5 mg (and if noncomplete response after 6 weeks, add-on mirabegron 25 mg).
3. Treatment group 2A: Mirabegron 25 mg (and if noncomplete response after 6 weeks, add-on mirabegron 25 mg).
4. Treatment group 2B: Mirabegron 25 mg (and if noncomplete response after 6 weeks, add-on solifenacin 5 mg).

**Figure 2:**
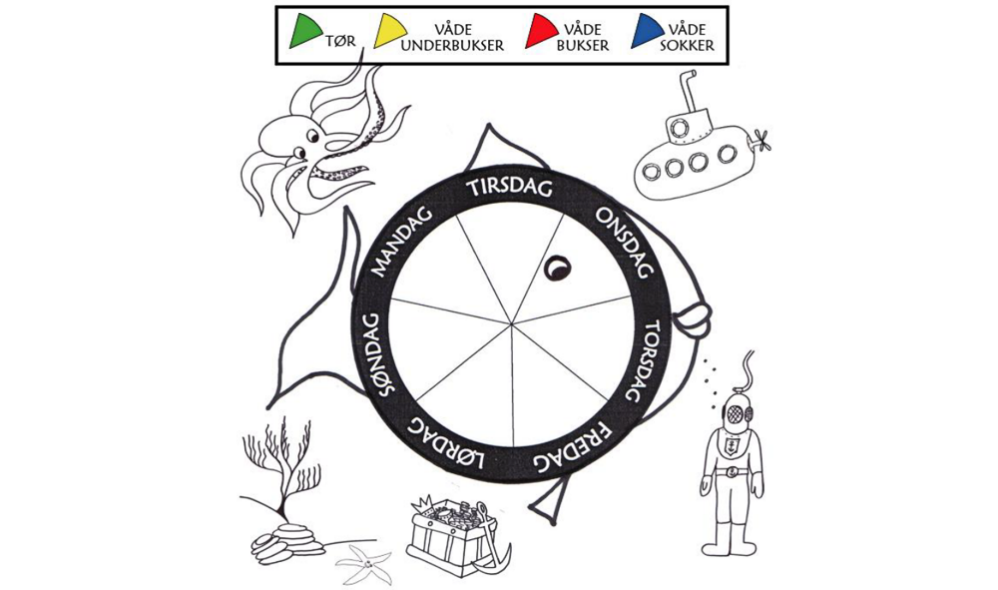
Danish translation of DryPie, developed by Bower et al [15]. Green: dry; yellow: wet underpants; red: wet trousers; blue: wet socks; Mandag: Monday; Tirsdag: Tuesday; Onsdag: Wednesday; Torsdag: Thursday; Fredag: Friday; Lørdag: Saturday; Søndag: Sunday.

All participants will initially receive low dose monotherapy. Two groups will receive solifenacin 5 mg and 2 groups will receive mirabegron 25 mg. After 6 weeks, the treatment outcome will be evaluated based on the number of wet days per week assessed by DryPie [15] (see Figure 2). Noncomplete responders will receive add-on treatment according to their primary randomization group; group 1A will receive solifenacin 5 mg and add-on solifenacin 5 mg, group 1B will receive solifenacin 5 mg and add-on mirabegron 25 mg, group 2A will receive mirabegron 25 mg and add-on mirabegron 25 mg, and group 2B will receive mirabegron 25 mg and add-on solifenacin 5 mg. Participants who show complete response within 6 weeks of treatment will continue the initial low dose monotherapy for the rest of the study period. Total treatment period will be 18 weeks.

### Withdrawal of Participants and Criteria for Discontinuation of Study

A study participant should be withdrawn from the study if at any time one of the following criteria is met:

1. It is the wish of the participant (or their parents/legally acceptable representative) for any reason (withdrawal of informed consent).
2. The discretion of the investigator for safety or behavioral reasons.
3. Severe noncompliance with protocol as judged by the investigator.
4. The investigator judges it necessary due to medical reasons.
5. Female participants who become pregnant should immediately discontinue study drug administration and will be discontinued from the study (female participants with childbearing potential are excluded from the study).
6. The participant experiences adverse reactions, which endanger the health of the participant, require emergency treatment, are incompatible with the continuation of the study, or are expected to influence the results of the study.
7. Participants with a condition or a situation that, in the investigator’s opinion, may put the participant at significant risk or may interfere significantly with the participant’s participation in the study will be discontinued from the study.
8. Participant with any other condition or situation that in the investigator’s opinion may be valid for discontinuation from the study.

### Primary End Point

The primary end point measure is treatment response assessed by change from visit 2 to end of study, according to the number of wet days per 7 days by DryPie.

### Secondary End Points

Secondary outcome measures change from baseline across the 18-week treatment period in the following parameters.

1. Treatment response, according to the number of wet days per 7 days by DryPie.
2. Change in incontinence severity score per 7 days assessed by DryPie.
3. Change in urge severity quantified by Bower Visual Analog Scale (VAS) Urgency Scheme [15].
4. Change in volume of maximum voided volume.
5. Change in volume of age-standardized maximum voided volume.
6. Change in volume of average voided volume.
7. Change in micturition frequency.
8. Change in total score of pediatric incontinence questionnaire (PinQ).
9. Change in total score of World Health Organization-Five Well-Being Index (WHO-5).

### Safety Outcomes

Safety outcome measures encompass the following.

1. Adverse event (AE), serious adverse event (SAE), and Suspected Unexpected Serious Adverse Reaction (SUSAR) monitoring.
2. Identifications of electrocardiogram abnormalities before entering the study and after 6 weeks.
3. Increase in blood pressure and pulse beyond the 95th percentile.
4. Change in ultrasound-assessed postvoid residual (PVR) urine from baseline to 18 weeks.
5. Identification of urinary tract infection by urine dipstick and verified by routine urine culture.

### Procedures During Pharmacotherapy

The participant timeline is illustrated in Table 1.

**Table 1:** Schedule of the assessments.

	Visit 1: baseline	Visit 2	Visit 3: end of study or premature discontinuation
Weeks (days)	0	6 (42)	18 (126)
Visit scheduling window (days)		(SD 5)	(SD 5)
Informed consent	✔		
Inclusion and exclusion criteria	✔		
Background information including demographics	✔		
Registration of concomitant medication	✔	✔	✔
Urotherapy	✔	✔	✔
Randomization	✔		
Dispensation of study medication	✔	✔	
Compliance		✔	✔
Drug accountability		✔	✔
Registration of side effects as reported in the product summary		✔	✔
Registration of adverse events/serious adverse events/suspected unexpected serious adverse reaction		✔	✔
Clinical examination	✔		
Weight and height	✔		
DryPie	✔	✔	✔
48-hour frequency-volume chart	✔	✔	✔
Bower Visual Analog Scale Urgency	✔	✔	✔
Uroflowmetry	✔		✔
Postvoid residual urine	✔	✔	✔
Blood pressure and pulse	✔	✔	✔
Electrocardiogram	✔	✔	
Urine dipstick test	✔	(✔)^a^	(✔)^a^
Pediatric Incontinence Questionnaire	✔		✔
World Health Organization-Five Well-Being Index	✔		✔

^a^Performed in case of symptoms of urinary tract infection.

### Visit 1 (Day 0/Week 0)

Participants will enter the study after parents/parental custody holders have signed the informed consent. The participant and parents will be instructed in filling in a 48-hour frequency-volume chart (one filled out in the last 2 months prior to visit 1 is accepted) and DryPie for 1 week prior to visit 1. Background information (including age, gender, DUI history, urotherapy, any concomitant medication present and previously, any other diseases, or psychiatric diagnosis) will be obtained. A clinical examination, including anthropometric measurements, measurement of blood pressure and pulse, uroflowmetry (or one performed the last 6 months prior visit 1) with ultrasound-assessment of PVR urine (or one performed the last 2 months prior visit 1), a urine dipstick, and an electrocardiogram, will be performed. Moreover, Bower VAS Urgency will be filled out. The quality of life and well-being of the participating children will be assessed using PinQ and WHO-5. The questionnaires will be filled out electronically at the outpatient clinic or at home with help from the parents or parental custody holders. Participants will be instructed in continuing urotherapy during the study. The participant will be randomized 1:1:1:1 to one of the 4 treatment groups described above. The participants will be unblinded and receive study medications according to their unique participant number. An unblinded study nurse can guide if necessary.

### Visit 2 (Day 42/Week 6)

The participant will be instructed to bring a 48-hour frequency-volume chart and the DryPie for 1 week, filled in prior to visit 2. Moreover, Bower VAS Urgency will be filled out during the visit. A clinical examination, including measurement of blood pressure and pulse, ultrasound-assessment of PVR urine, safety electrocardiogram, and a urine dipstick test (in case of symptoms of urinary tract infection), will be performed. Registration of side effects as reported in the product summaries will be performed. Any AE, SAE, or SUSAR will be registered and handled according to Good Clinical Practice.

The treatment outcome will be evaluated based on the number of wet days per week assessed by DryPie. Based on the International Children’s Continence Society’s definitions of treatment outcome, the participants in group 1 (solifenacin 5 mg) and group 2 (mirabegron 25 mg) will be treated as follows.

1. Level 1: If complete response (100% reduction in number of wet days), then maintain current treatment level.
2. Level 2: If not complete response (<100% reduction in number of wet days), then treatment 1A group receives solifenacin 5 mg + solifenacin 5 mg, treatment 1B group receives solifenacin 5 mg + mirabegron 25 mg, treatment 2A group receives mirabegron 25 mg + mirabegron 25 mg, and treatment 2B group receives mirabegron 25 mg + solifenacin 5 mg.

The dose titration will be notified in the electronic patient journal, and as the study is blinded by investigators, it will only be noted whether the participant is treated with study medication level 1 or level 2. An unblinded nurse will guide and answer questions to participants in case of dose titration. The compliance to the study medication will be evaluated by an unblinded project nurse at every site. Compliance will be evaluated by counting the remaining tablets in the container on every visit. Three categories will be used: ≥80%, 50%-79%, or <50%.

The participant should contact the outpatient clinic by phone between visit 2 and visit 3 in case of any AE, SAE, or SUSAR. In case dose titration to level 2 results in increased PVR urine, symptoms of urinary tract infections, positive urine dipstick, side effects, or development of hypertension, the investigator can adjust the dose to the initial dose (level 1).

### Visit 3 (Day 126/Week 18)

The participant will be instructed to bring a 48-hour frequency-volume chart and the DryPie for 1 week, filled in prior to visit 3. Moreover, Bower VAS Urgency will be filled out during the visit. A clinical examination, including measurement of blood pressure and pulse and ultrasound-assessment of PVR urine, will be performed. In case of symptoms of urinary tract infection, a urine dipstick test will be performed. Side effects will be registered as reported reference documents of side effects, as reported in the product summaries. Any AE, SAE, or SUSAR will be registered and handled according to Good Clinical Practice. The quality of life at the end of the treatment will be evaluated using PinQ and WHO-5. The compliance to the study medication will be evaluated by an unblinded project nurse at every site by counting the remaining tablets in the container. At the end of the study or in case of premature discontinuation, the child continues at the outpatient clinic at the respective study site to determine the future treatment. An unblinded medical doctor at the study site will decide the future treatment.

### Randomization Procedure

Randomization will be performed in the REDCap (research electronic data capture) portal and will occur centrally. An electronic case report form will be built for the study data to be entered. The randomization will be performed in a separate module, and the randomization list will be maintained by the unblinded nurse at the Department of Pediatric and Adolescent Medicine, Gødstrup Hospital, until the end of the study.

Following written informed consent, randomization is stratified by 1:1:1:1 allocation within each stratum by using predefined block sizes for sites. Block randomization is by a computer-generated random number list. The investigators and all other medical staff are kept blinded to the allocation, except for the unblinded study nurses. The unblinded study nurse at each study center will have a unique account for the electronic case report form that allows randomization and seeing which product needs to be prepared for the study participant. The blinded study team will not be able to see the allocation in the electronic case report form. The participants will be unblinded and receive study medication according to their unique participant number.

### Blinding and Unblinding

This study is single-blinded, and the study participant will receive medications delivered from the study site. The investigator does not know which treatment the individual participant receives. Participants will be informed that they are not allowed to reveal the study medication to the blinded investigator, and an unblinded nurse will guide and answer questions to the participants in case of dose titration. The children and their parents will be asked for possible side effects at every visit and will always be able to contact a medical doctor or an investigator when a side effect is suspected. The participant should contact the outpatient clinic by phone between visit 2 and visit 3 in case of any AE, SAE, or SUSAR. All participating children will be provided with a trial card on which the investigational product, the trial number, investigator’s name, and a 24-hour emergency contact number are stated. Unblinding is possible in case of an SAE or SUSAR. If an investigator needs to unblind a study participant due to a safety issue, the investigator will be able to do this in REDCap. Unblinding will be performed according to Good Clinical Practice guidelines. Any unblinding will be logged per user, with a timestamp. The investigator will keep the rest of the study team and study participant blinded, unless the nature of emergency requires that all parties should be informed. The sponsor will be notified of the unblinding.

### Statistical Analysis

The primary outcome will be analyzed as follows. First, the change from visit 2 across the 18-week pharmacological treatment period in the number of wet days per week assessed by DryPie will be assessed for all the groups. Second, treatment response (either nonresponse or response) will be assessed for all the groups. Third, to analyze and compare the treatment groups, the Pearson chi-square test will be used. The secondary outcomes of treatment response across 18 weeks will be analyzed as follows. First, change from baseline across the 18-week pharmacological treatment period in the number of wet days per week assessed by DryPie will be assessed for all the groups. Second, treatment response (either nonresponse or response) will be assessed for all the groups. Third, to analyze and compare the treatment groups, the Pearson chi-square test will be used. Additionally, secondary outcomes will be reported as follows. Continuous data will be reported as median with interquartile range for nonnormally distributed data and as mean (SD) for normally distributed data, and the assumption of normally distributed data will be checked by Q-Q plots. Categorical data will be presented as number (%). Safety parameters (treatment-emergent AEs, SAEs, vital signs, electrocardiogram, PVR urine volume, urinary dipstick) will be summarized using descriptive statistics.

### Sample Size

The estimates for the sample size calculations are based on 1 placebo-controlled trial, prospective studies, and retrospective studies [10-12,14,16]. We expect 15% of the children to have a complete response to treatment with low dose solifenacin 5 mg and 15% of the children to have a complete response to treatment with low dose mirabegron 25 mg. Of the remaining children (100% – 15% = 85%) who are noncomplete responders to low dose monotherapy, we expect 20% to have complete response to solifenacin 10 mg, 20% to have complete response with mirabegron 50 mg, and 50% to have complete response to combination therapy (solifenacin and mirabegron).

With the abovementioned estimations, we assessed a proportion of 32% (15% with complete response to solifenacin 5 mg + 85% noncomplete responders to low dose monotherapy solifenacin 5 mg × 20% with complete response to solifenacin 10 mg) of the participants randomized to solifenacin 10 mg will have a complete response. In addition, we assessed a proportion of 57.5% of the participants randomized to solifenacin 5 mg and mirabegron 25 mg. The sample size calculation was based on solifenacin 10 mg versus combination therapy and mirabegron 50 mg versus combination therapy. The estimated sample sizes for a 2-sample proportions test were calculated by Pearson chi-square test. With a power of 80% and for obtaining a statistically significant (α=.05) difference between solifenacin 10 mg and combination therapy of solifenacin 5 mg and mirabegron 25 mg, the sample size was calculated by a 2-sample proportions test.

To obtain a power of 80% and with 2-sided statistical significance level of 5%, given the probability of complete response to solifenacin 10 mg is 32% (π_1_=0.32) and the probability of complete response to combination therapy of solifenacin 5 mg and mirabegron 25 mg is 57.5% (π_2_=0.575), the required sample size is 59 in each group (1A, 1B, 2A, 2B). The sample size included the expected children who will continue the initial dose. In total, the required sample size is 236 participants.

### Ethics Approval

This study is authorized by The Danish Medicines Agency and The Danish Health Research Ethics Committee and registered at Clinical Trials in the European Union (EU CT 2023-510187-13-00).

### Ethical Considerations

All pharmacological side effects will be handled in accordance with the Danish legislation. No risk or unknown side effects are expected from urotherapy, medical treatment, or withdrawal. No risks are expected by clinical examination and paraclinical measurements. The therapeutic potential for future patients justifies the project to be performed. Participation in this study will not lead to any disadvantages for the patient in their treatment. This study will be conducted in accordance with the protocol, applicable regulatory requirements according to Good Clinical Practice, and the ethical principles of the Declaration of Helsinki. Information regarding the participants is protected and anonymized according to the General Data Protection Regulation and the actual law. The study participants and their families will not be economically compensated for participating in this study; however, study medication costs will be covered throughout the entire study period.

### Safety Evaluations

Safety evaluations include AE, SAE, and SUSAR monitoring, registration of side effects as reported in the product summary, blood pressure, pulse, ultrasound-assessed PVR, and identification of urinary tract infection by urine dipstick and verified by routine urine cultivation and electrocardiogram. The listed safety parameters, except electrocardiograms, will be monitored at every visit during the study treatment administration, and urine dipstick will be performed at visit 2 and 3 if a participant reports symptoms of a urinary tract infection. Electrocardiograms will be monitored at baseline and at visit 2. The children and their parents will be asked for possible side effects at every visit and will always be able to contact a medical doctor or an investigator when a side effect is suspected. All participating children will be provided with a trial card on which the investigational product, trial number, investigator’s name, and a 24-hour emergency contact number are stated.

## Results

The BeDry study was approved by Clinical Trials in the European Union on June 12, 2024. Recruitment began on June 27, 2024, and will continue until 236 patients are included, which is expected to occur by September 2026. As of April 2025, 75 participants are included. The results will be submitted to Clinical Trials in the European Union and ClinicalTrials.gov within 1 year after the end of the trial.

## Discussion

### Anticipated Findings and Importance of This Research

Solifenacin is approved for the treatment of neurogenic detrusor overactivity in children and adolescents aged 2-18 years [17,18]. Though not approved for the treatment of idiopathic OAB in children, solifenacin is widely used off-label. The efficacy of solifenacin was found superior to placebo in terms of average voided volume per miction, daily maximum voided volume, and micturition frequency adjusted for total voided volume [11]. The efficacy of solifenacin by decreasing number of incontinence episodes per day was demonstrated by non–placebo-controlled prospective studies [19,20]. The safety and tolerability of solifenacin were found to be high, also by long-term use [12]. Predominantly, side effects such as xerostomia, constipation, blurred vision, headache, and fatigue were reported [11,19,20]. A few participants experienced more severe side effects, encompassing QT prolongation, severe constipation, fecal incontinence, and aggressive behavior, some of whom withdrew from the study due to the intolerable side effects and for safety reasons.

Mirabegron has been approved as an alternative to antimuscarinics in children with neurogenic detrusor overactivity [21]. In the pediatric population, mirabegron is used off-label in children with OAB, either as monotherapy or in combination with antimuscarinics [13,14]. The efficacy of mirabegron compared to that of placebo has been elucidated among children [10]. Furthermore, mirabegron has high safety and tolerability in the pediatric population [22]. Reported side effects [22] were blurred vision, constipation, abdominal cramps, and these were transient. No additional risk to the study participants is anticipated with the use of solifenacin and mirabegron.

A favorable safety profile was observed. In adult populations, the efficacy benefits of solifenacin and mirabegron outweigh the risks. This favorable benefit-risk ratio supports the further development of solifenacin and mirabegron in the treatment of pediatric populations with DUI.

With our protocol, we aim to investigate if treatment with a combination of solifenacin and mirabegron in low dose is effective in the reduction of DUI episodes over an 18-week treatment period compared to baseline and if combination therapy in low dose is superior to maximum dose of either solifenacin or mirabegron in children with noncomplete response to urotherapy and low dose monotherapy of solifenacin or mirabegron.

The results from our study will ensure evidence-based knowledge about the off-label treatment approach. By optimizing the stepwise medical treatment approach, the treatment can be shortened, side effects can be diminished, and unnecessary medical expenses can be minimized for individual families and the society both nationally and internationally.

Compared to experience from daily practice, randomized controlled trials allow for the systematic evaluation of both the safety and efficacy of treatments. This is crucial in pediatrics, where considerations such as appropriate dosing, potential side effects, and long-term outcomes need to be carefully assessed to ensure the well-being of children receiving treatment.

### Strengths and Limitations

The strengths of our study are the randomized single-blinded and controlled design with multicenter inclusion at 5 pediatric departments in Denmark. The investigators are blinded, and the participants know the treatment. This could cause a possible risk of affecting the final outcome of the study. Hence, no placebo effect is introduced. With single blinding, the investigators are not biased while treating the patients nor when interpretating data.

This study is embedded into daily clinical practice and reflects standard practice in many aspects other than randomization and collection of data. Usually, mirabegron is add-on pharmacotherapy if anticholinergics are not fully effective in treating DUI. The pragmatic design ensures timely inclusion of patients and reflects daily clinical practice where pharmacotherapy is initiated after inadequate effects of urotherapy.

The main limitation is compliance to the study medication over 18 weeks. We aim to reduce this risk by early termination of children with severe compliance issues (as <50%) during the treatment period. Compliance to the study medication is determined by counting the remaining tablets in the containers at each visit. This is performed by an unblinded nurse.

The risk of unblinding the blinded investigators is present, but we expect to reduce the risk by introducing unblinded study staff as project nurses, as well as the participants and their parents are instructed to not inform the investigator about the medication. Another limitation is the fixed dose of the combination therapy, thereby introducing a potential risk of undertreating the children allocated to the intervention group.

### Conclusion

If the low dose of combination therapy is superior to the high dose of monotherapy, children with DUI will benefit significantly from the new treatment in terms of shorter treatment periods, fewer side effects, and possibly a better quality of life. This trial has the potential to optimize the medical treatment approach, shorten the treatment period, diminish the side effects, and minimize unnecessary medical expenses. Due to predefined criteria for discontinuation of the allocated therapy, we expect the risk of treatment failure to be minimal. The results are expected to influence the treatment strategy of children with DUI worldwide.

## Appendix

Multimedia Appendix 1 CONSORT (Consolidated Standards of Reporting Trials) checklist.

## Abbreviations

AE: adverse event
CONSORT: Consolidated Standards of Reporting Trials
DUI: daytime urinary incontinence
OAB: overactive bladder
PinQ: pediatric incontinence questionnaire
PVR: postvoid residual
REDCap: research electronic data capture
SAE: serious adverse event
SUSAR: suspected unexpected serious adverse reaction
VAS: Visual Analog Scale
WHO-5: World Health Organization-Five Well-Being Index

## Data Availability

The data sets generated during this study are planned to be available on reasonable request.
